# An Economical Pudding

**Published:** 1886-01

**Authors:** 


**An Economical Pudding.**—A very nice and economical pudding is made by bruising, through a colander, six large or twelve small sized boiled potatoes ; beat four eggs, mix with a pint of milk, stir in the potatoes, sugar and seasoning to taste ; butter a dish ; bake half an hour.

